# Sequential Growth of Uniform β-NaYF_4_@β-NaLnF_4_ (Ln = Y, Lu, Yb) Microcrystals with Luminescent Properties of Multicolor Tuning and Dual-Mode Emission

**DOI:** 10.3390/nano7120448

**Published:** 2017-12-14

**Authors:** Dandan Ju, Feng Song, Yingdong Han, Wenjing Cui, Aihua Zhou, Shujing Liu, Xueqin Wang, Ming Feng, Chengguo Ming

**Affiliations:** 1 School of Physics, Nankai University, Tianjin 300071, China; judandan@mail.nankai.edu.cn (D.J.); nkuhanyd@mail.nankai.edu.cn (Y.H.); 1120130060@mail.nankai.edu.cn (W.C.); zhouaihua@mail.nankai.edu.cn (A.Z.); wangxueqin@mail.nankai.edu.cn (X.W.); mingfeng@nankai.edu.cn (M.F.); 2 The Collaborative Innovation Center of Extreme Optical, Nankai University, Tianjin 300071, China; 3 School of Biomedical Engineering, Tianjin Medical University, Tianjin 300070, China; liushujing@tijmu.eud.cn; 4 School of Science, Tianjin University of Science & Technology, Tianjin 300457, China; mingchengguo@tust.edu.cn

**Keywords:** NaYF_4_ microcrystals, microscale core-shell structures, multicolor tuning, dual-mode emission

## Abstract

We synthesized the uniform core-shell microstructured compounds with hexagonal phase NaYF_4_:Er/Yb microrods as the core and hexagonal phase NaLnF_4_ (NaYbF_4_, NaLuF_4_:Yb/Tm, NaYF_4_:Yb/Er, NaYF_4_:Eu) as the shell based on the hydrothermal reaction. These microscale core-shell structures provided a platform for the spatially confining optical process while possessing high luminescence efficiency. The thickness of the shell could be controlled by adjusting the amounts of shell precursor, which significantly affected the intensity of the shell dopant ions emission and the emission color of core-shell upconversion luminescence (UCL). The uniform NaYF_4_@NaLnF_4_ (Ln = Y, Lu, Yb) microrods, with a series of rare-earth ions doped into the core and shell layer at various doping concentrations, achieved color-tuning of the upconversion (UC) emission and dual-mode emission at the single-microcrystal level, thus allowing the efficient utilization of core-shell microcrystals in the photonics and security labeling. This study suggests a new class of luminescent materials in the microscopic field.

## 1. Introduction

Lanthanide-doped UC materials always attract wide attention due to their special anti-Stokes shifting, which allow emitting visible light under near-infrared (NIR) light excitation [1,2,3,4]. Compared with organic dyes or quantum dots, the UC materials have significant advantages, such as long luminescent lifetime, narrow emission band, and low cost [5,6]. The UC materials can be applied in many fields, including biomarkers, display, security printing, solar cells, photodynamics therapy (PDT), and temperature sensing [7,8,9,10,11,12,13,14,15,16,17,18,19]. However, the utilization of traditional UC materials is limited by their low luminescence efficiency and intractable emission color [1,6,7,20,21,22,23,24,25]. The significant improvement in the luminescence efficiency and multicolor emission achievement had been realized through the growth of inert/active shells around the core, such as NaYF_4_:Yb/Er@NaLnF_4_:Yb/Tm@NaGdF_4_ (Ln = Y, Yb, Lu, Gd) core-multishell nanoparticles, which can protect the lanthanide ions in the core from nonradiative decay and control the energy transfer of different lanthanide ions for multicolor emission [26,27,28,29,30]. Therefore, the NaLnF_4_@NaLnF_4_ (Ln = Y, Yb, Lu, Gd) based core-mutishell nanoparticles have drew extensive attention. As for nanoscale structure, an inert shell as the outermost layer is always required to enhance the emission intensity by decreasing the surface quenching effects [31,32]. However, the additional step increases the complexity of the process and failure rate. The NaLnF_4_ (Ln = Y, Yb, Lu, Gd) based core-shell upconversion microcrystals can conveniently achieve multicolor emission even at a single microcrystal level due to their microscale dimensions. 

Despite the reported core-shell microcrystals with uniform nonepitaxial layers [33,34], Liu et al. had reported epitaxial growth of core-shell microcrystals through controlling the growth orientation along specific crystal faces [35,36]. The core-shell structured microcrystals whose growth orientations are along all crystal faces, have been scarcely reported. Thus, it is necessary to further explore the core-shell epitaxial microstructure with a series of rare-earth ions doped into the core and shell layer, respectively. It is of great importance to develop a suitable strategy to synthesize the core-shell upconversion microcrystals. 

In this study, we presented the preparation of the uniform core-shell structured β-NaLnF_4_@β-NaLnF_4_ (Ln = Y, Lu, Yb) microcrystals. The core-shell structures and chemical composition distribution on core/shell microcrystal were obtained by scanning transmission electron microscopy (STEM) and energy dispersive X-ray spectroscopy (EDS). We realized the multiple colors (red/green/blue) in these core-shell microcrystals by 980 nm laser excitation at a single microrod level. Meanwhile, the core-shell (β-NaYF_4_:Yb/Er@NaYF_4_:Eu) microrods achieved independent dual-mode emission under near-infrared (NIR) and ultraviolet (UV) excitations. The core-shell structure of microcrystals can serve as ideal candidates in various fields, such as in photonics and anti-counterfeiting detection.

## 2. Results and Discussion

In this section, we first confirm the core-shell structure of upconversion microcrystals by STEM and EDS analysis, followed by a discussion of the microcrystals with different shell thicknesses by adjusting the amount of the shell precursor. Next we describe the upconversion emission of the core-shell microcrystals with different shell thicknesses. The final section presents the applications of these UC microcrystals, such as color tuning and dual-mode emission.

### 2.1. Confirmation of the Core-Shell Structure of Upconversion Microcrystals

**Figure 1:**
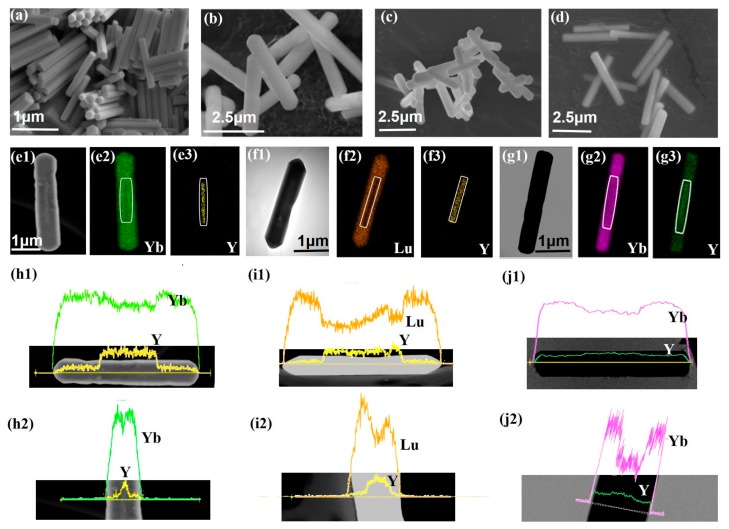
(**a**–**d**) SEM images of the microcrystals, NaYF_4_:Yb/Er (20/2 mol %, Y), NaYF_4_:Yb/Er (20/2 mol %)@NaYbF_4_, NaYF_4_:Yb/Er (20/2 mol %, Y)@NaLuF_4_:Yb/Tm (20/0.2 mol %) and NaYF_4_:Yb/Er (20/2 mol %, Y)@NaYF_4_:Yb/Er (80/2 mol %, R); (**e1**–**e3**) Scanning transmission electron microscopy image (STEM) of the NaYF_4_:Yb/Er (Y)@NaYbF_4_ microrods and element mappings of Yb and Y in a single core-shell microrod; (**f1**–**f3**) STEM image of the NaYF_4_:Yb/Er (G)@NaLuF_4_:Yb/Tm microrods and element mappings of Yb and Y in the core-shell microrod shown in figure (**c**); (**g1**–**g3**) STEM image and element mappings of Yb, Y in a single core-shell microrod (NaYF_4_:Yb/Er@NaYF_4_:Yb/Er). The white boxes show the position of the core microrods in (**e**–**g**); (**h1**–**j2**) Line scans of the elemental distribution in NaYF_4_:Yb/Er (20/2 mol %)@NaYbF_4_, NaYF_4_:Yb/Er (20/2 mol %, Y)@NaLuF_4_:Yb/Tm (20/0.2 mol %) and NaYF_4_:Yb/Er (20/2 mol %, Y)@NaYF_4_:Yb/Er (80/2 mol %, R) microrods, respectively.

The core-shell NaLnF_4_@NaLnF_4_ microcrystals were fabricated by an epitaxial growth process (see Figures S1–S3 in the Supplementary Information). Figure 1a shows the scanning electron microscopy (SEM) image of the NaYF_4_:Yb/Er (20/2 mol %) seeding crystals, which indicates the uniform upconversion morphology of these microcrystals. The length of the obtained seed is about 1.38 μm. Figure 1b–d shows the SEM images of core-shell structured β-NaYF_4_:Yb/Er@β-NaYbF_4_, NaYF_4_:Yb/Er@NaLuF_4_:Yb/Tm and NaYF_4_:Yb/Er@NaYF_4_:Yb/Er microcrystals, respectively. The evolution of particle size clearly indicates the successful epitaxial growth of NaLnF_4_ (Figure S4a,b). For example, in Figure 1b, the microrods have a length of about 3.0 μm and a diameter of about 0.42 μm. Figure 1(e1) displays the STEM images of a typical NaYF_4_:Yb/Er@NaYbF_4_ microrod and the core (bright) can be easily distinguished from the shell (dark) materials. The brightness difference is caused by the larger atomic number of Yb (*Z* = 70) compared with Y (*Z* = 39) [37,38]. The core has the same length and diameter with the seed. Corresponding EDS mappings of the Yb, Y of STEM images are shown in Figure 1(e2,e3). Figure 1(h1,h2) shows the line scans across the single microrod along the axial direction and radial direction, respectively. It is obvious that the intensity of element signals varied with the position of the samples. The intensity of the Y signal at the center of the microrods is significantly larger than that at the edge. The intensity distribution of the Y signal is opposite to that of the Yb signal. STEM and EDS analysis illustrate that the chemical composition distribution of the microcrystals is consistent with the designed element distribution (Figure S4c–k), indicating the formation of core-shell structure (Figure S4). In addition, the prepared core-shell structured microcrystals are pure hexagonal phase (Figure S4l). The preparation approach of core-shell microcrystals is also applicable to the core-shell structure with an active shell layer, such as NaYF_4_:Yb/Er@NaLuF_4_:Yb/Tm, NaYF_4_:Yb/Er (Y)@NaYF_4_:Yb/Er (R) (Figure 1f,g,i,j and Figures S5 and S6).

### 2.2. Core-Shell Microcrystals with Different Shell Thicknesses

**Figure 2:**
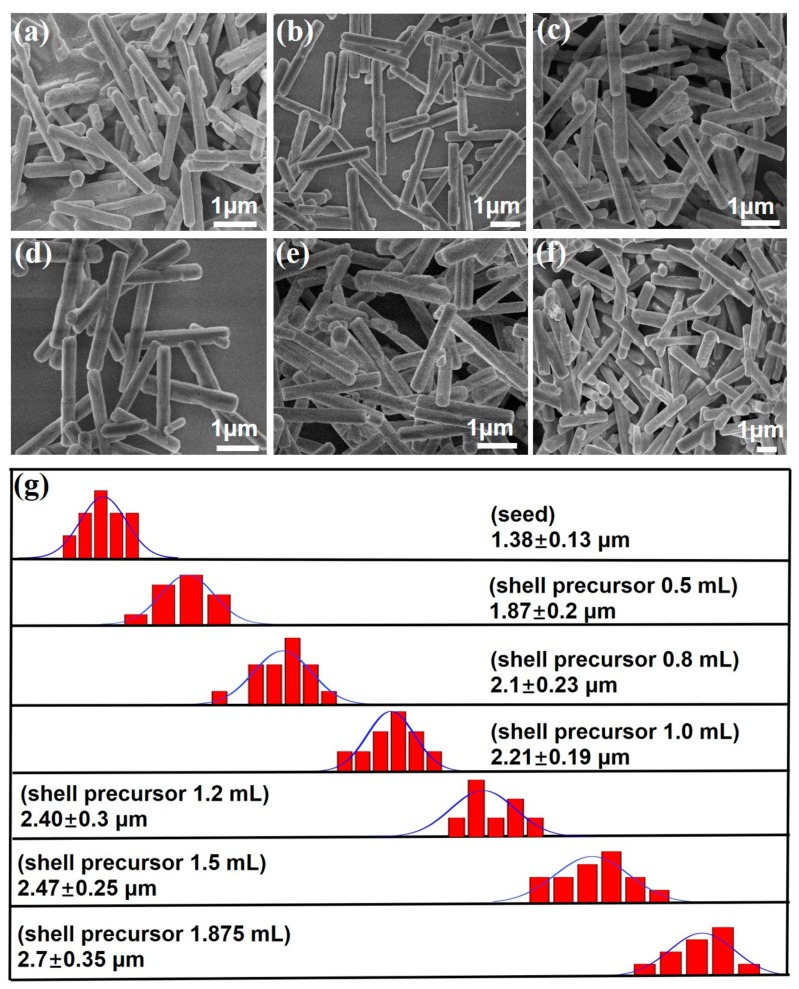
Investigation of NaYF_4_:Yb/Er (20/2 mol %, Y)@NaLuF_4_:Yb/Tm (20/0.2 mol %) microcrystals growth versus the amount of shell precursor. (**a**–**f**) are the SEM images of the core-shell microcrystals with different shell thicknesses, the volume of shell precursor: (**a**) 0.5 mL; (**b**) 0.8 mL; (**c**) 1.0 mL; (**d**) 1.2 mL; (**e**) 1.5 mL; (**f**) 1.875 mL; (**g**) Size distribution analysis of the NaYF_4_:Yb/Er (20/2 mol %, Y)@NaLuF_4_:Yb/Tm (20/0.2 mol %) microcrystals collected with different shell thicknesses.

The core-shell microcrystals with different shell thicknesses were obtained by adjusting the amount of the shell precursor. As shown in Figure 2 and Figure S7, the samples exhibit the uniform size distribution. After increasing the shell precursor solution from 0.5 mL to 1.875 mL (2 M), the length of the β-NaYF_4_:Yb/Er@β-NaLuF_4_:Yb/Tm microcrystals increased from 1.87 μm to 2.70 μm and the diameter of microcrystals increased from 0.3 μm to 0.42 μm (Figure S8). The findings provide a convenient method to achieve the tunable shell thickness, thus allowing effectively manipulating of the upconversion color of the microcomposite.

### 2.3. UC Luminescence Properties of Core-Shell Structured Microcrystals

**Figure 3:**
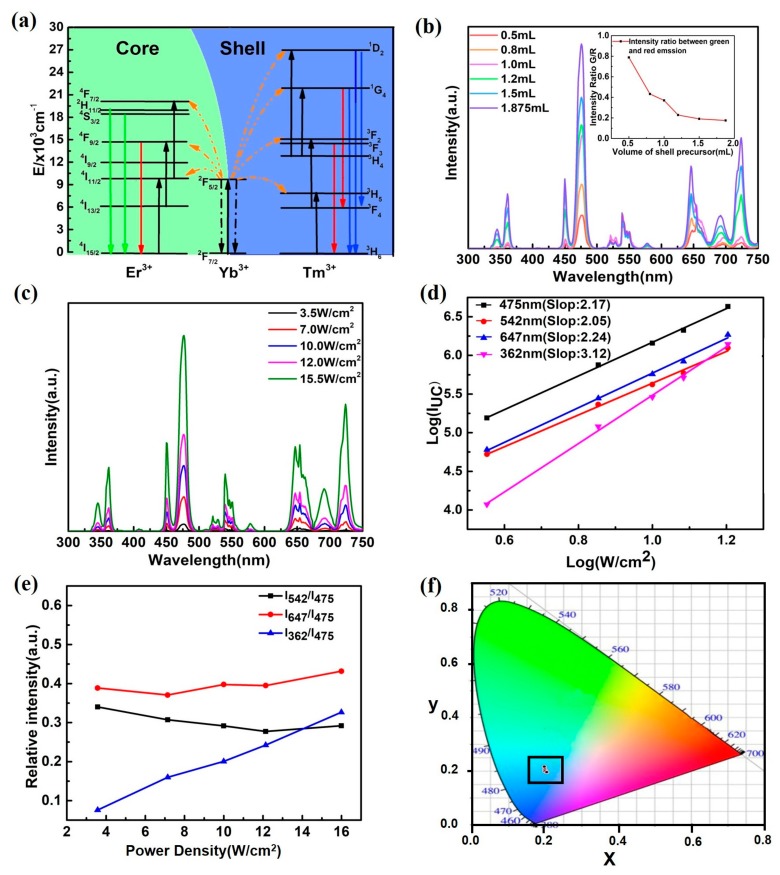
(**a**) Schematic diagram of the energy levels of Er^3+^, Yb^3+^, and Tm^3+^ and UC progresses under 980 nm laser excitation; (**b**) UCL spectra of the core-shell microcrystals with different volumes of shell precursor under the irradiation of 980 nm (~10 W/cm^2^) (red: 0.5 mL; orange: 0.8 mL; yellow: 1.0 mL; green: 1.2 mL; cyan: 1.5 mL and blue: 1.875 mL); The inset of (**b**) shows the G/R ratio as the function of the volume of shell precursor; (**c**) pump-power-dependent UCL emission spectra of core-shell structured microcrystals (with both Er^3+^ and Tm^3+^ character emissions); (**d**) log–log plots of UC emission intensity versus excitation power density; (**e**) relative intensity of different emission bands under different irradiation (black, red and blue lines represent core-shell microcrystals); (**f**) International Commission on Illumination (CIE) chromaticity coordinates of the emission for different excitation power density.

We further investigated the upconversion emission of the core-shell microcrystals with different shell thicknesses. Figure 3a shows the energy level diagram of Yb^3+^, Er^3+^, and Tm^3+^ and the upconversion mechanisms for the β-NaYF_4_:Yb/Er@β-NaLuF_4_:Yb/Tm system. The strong emission bands of core microrods centered at 523 nm, 542 nm and 647 nm correspond to H11/22→I15/24, S3/22→I15/24, and F9/24→I15/24 processes, respectively. The emission peaks of shell at 362 nm, 450 nm, 475 nm, 646 nm and 696 nm derive from the transition of D21→H63, D21→F43, G41→H63, G41→F43, and F33→H63.

The thickness of the shell in our core-shell microcrystals has a great influence on the emission intensity of the shell dopant ions (Tm^3+^) emission, as shown in Figure 3b. Interestingly, the emission intensity of dopant ions in core (Er^3+^) does not change significantly with an increase in the shell thickness. In addition, compared with the intensity of the seeding microrods, the coating modification results in a decrease in the UCL intensity of the dopant ions in the core. Two mechanisms are proposed to explain the phenomenon. Firstly, the 980-nm excitation light is absorbed largely by the Yb^3+^ in the shell layer before arriving at the NaYF_4_:Yb/Er core [32,39]. Secondly, the shell layer is anisotropic for the core, which affects the polarization of UCL of hexagonal microcrystal and further induces the suppression of Er^3+^ emission [37,40,41]. The inset of Figure 3b shows that the green-to-red (G/R) intensity ratios with different volumes of shell precursor. The G/R ratio of β-NaYF_4_:Yb/Er@β-NaLuF_4_:Yb/Tm microcrystals with 0.5 mL shell precursor is about 4.5 times higher than that of the sample with 1.875 mL shell precursor (0.175). The green emission intensity remains nearly constant, and the red emission intensity is enhanced due to enhancement of luminescence intensity from Tm^3+^ ions, whose contents increase with increasing the volume of shell precursor. Consequently, G/R ratios decrease with increasing the volume of shell precursor.

Figure 3c shows the pump-power-dependent UCL emission spectra of core-shell structured microcrystals. The blue, green and red emissions of the core-shell structured microcrystals are increased by increasing the excitation power density. The log–log plots of emission intensity versus excitation power density are shown in Figure 3d, the slopes obtained were 3.12, 2.24, 2.05 and 2.17 for the 362 nm, 475 nm, 542 nm and 647 nm emission in core-shell structured microcrystals. This result means that four-, three- and two-photon process are all involved. The log–log plots of emission intensity versus excitation power density indicate that the plots of emissions peaks (475 nm, 542 nm and 647 nm) are nearly parallel (Figure 3d). Similar results are observed in NaYF_4_:Yb/Tm and NaYF_4_:Yb/Er microcrystals [41,42,43]. In other words, similar UC mechanisms are generated. The emission intensity at 362 nm increases obviously with the increase in the excitation power density (Figure 3e), indicating that the four photon processes in Tm^3+^ is promoted under high excitation power density. However, the relative intensities of blue (475 nm), green (542 nm) and red (647 nm) emissions are nearly constant at different excitation power densities (Figure 3e), indicating that the emission color is independent on the power density (Figure 3f).

### 2.4. Upconversion Color Tuning

**Figure 4:**
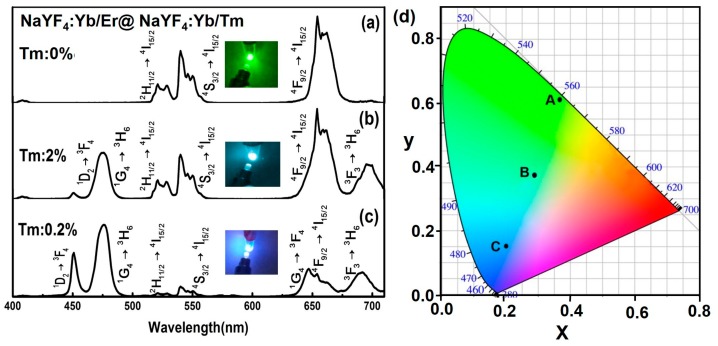
(**a**—**c**) UC luminescence spectra of the NaYF_4_:Yb/Er (20/2 mol %)@NaLuF_4_:Yb/Tm(20/*x* mol %, *x* = 0, 2, 0.2) with different concentrations of Tm^3+^ in the shell under 980 nm laser excitation (~15.5 W/cm^2^); (**d**) CIE chromaticity coordinates of the emission for different concentrations of Tm^3+^ (Tm^3+^: 0%, 2%, 0.2%) in the shell.

In full-color displays, multiplexed encoding and solar cells, it is required to precisely control the emission profiles of upconversion nanocrystals [1,9]. The conventional method for tuning the colors was through optimization of the type and concentration of Ln^3+^ ions (Yb, Er, Tm, Tb, Dy, and Eu) or developing the FRET (fluorescence resonance energy transfer) system with core/shell nanoparticles [44]. The core-shell microcrystals could also achieve multicolor tuning by controlling the types and concentrations of rare-earth ions under single-wavelength excitation, as shown in Figure 4.

Next, we investigated the multicolor upconversion emission and color tuning by adjusting the concentrations of activators (Tm^3+^) in-shell. In the β-NaYF_4_:Yb/Er@β-NaLuF_4_:Yb/Tm system, the concentration of Tm^3+^ in the shell layer can be altered to control the interaction among dopant ions, thus affecting blue emission intensity while maintaining the luminescence intensity of red and green bands generated by the core-shell structure. Figure 4a–c show the emission spectra tuned through Tm^3+^ doping in core-shell microrods (NaYF_4_:Yb/Er@NaLuF_4_:Yb/Tm) under 980-nm laser excitation. We demonstrated that the emission upconversion color tuning from green to blue could be achieved by doping activators of Er^3+^ and Tm^3+^ at specific concentration ratios in the core and shell layer (Figure 4d). From Figure 4d, we could obtain the positions of various samples with different concentrations of Tm^3+^ in CIE chromaticity coordinates. The A, B, C spots represent the coordinate (*X* = 0.3692, *Y* = 0.6105), (*X* = 0.2849, *Y* = 0.3739) and (*X* = 0.2013, *Y* = 0.1528) of NaYF_4_:Yb/Er (20/2 mol %)@NaLuF_4_:Yb/Tm(20/*x* mol %, *x* = 0, 2, 0.2), respectively. In conclusion, we achieved the color tuning by changing the concentration of activators in shell.

### 2.5. Dual-Mode Emission

**Figure 5:**
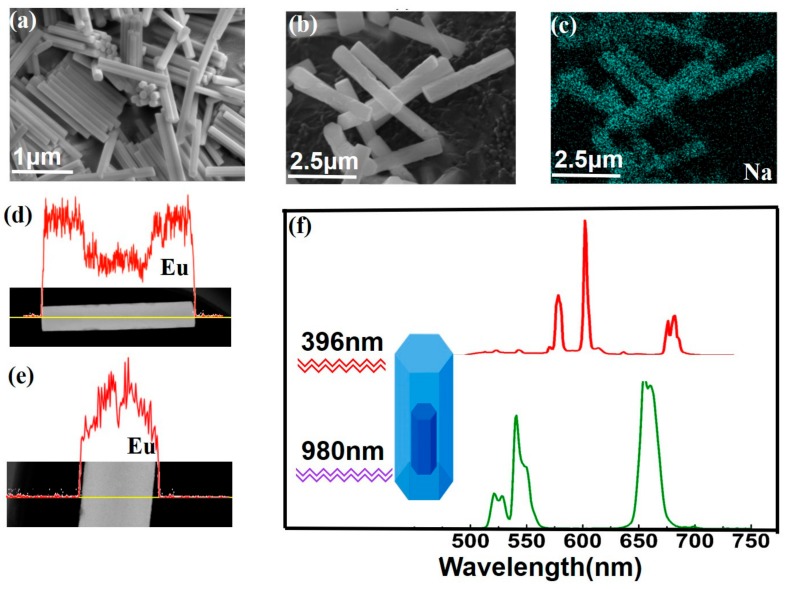
(**a**,**b**) SEM images of the microcrystals, NaYF_4_:Yb/Er (5/0.05 mol %) and NaYF_4_:Yb/Er (5/0.05 mol %)@NaYF_4_:Eu (10 mol %); (**c**) element mappings of Na in the microcrystals; (**d**,**e**) line scans of the elemental distribution in a single core-shell microrod; (**f**) emission spectra of the microcrystals under excitation at 396 nm and 980 nm, respectively. The excitation power density is about 15.5 W/cm^2^ for UC emission.

Dual-mode emission in a single particle extends the emission spectra of particles throughout an almost visible region by incorporating lanthanide ions (Yb^3+^, Er^3+^, Tm^3+^, Dy^3+^, Sm^3+^, Ho^3+^, Eu^3+^, and Tb^3+^) into the core or shell layer. In this paper, we selected the Er^3+^ ions and Yb^3+^ ions as dopants for the UC process and chose the Eu^3+^ ions as dopants for the UV-to-visible emission in β-NaYF_4_ host material. The uniform β-NaYF_4_:Yb/Er (5/0.05 mol %)@β-NaYF_4_:Eu (10 mol %) microrods were formed by the same method described above (Figure S9). The SEM images of seeds and core-shell samples are shown in Figure 5a,b. The sizes of seeds and core-shell are 1.4 ± 0.14 μm and 3.0 ± 0.35 μm, respectively. Figure 5c shows element mappings of Na, and Figure 5d,e display line scans of the elemental distribution across the single microrod along the axial direction and radial direction, respectively.

Through selecting the dopant concentrations, NIR-to-visible and UV-to-visible emission were combined together in a single core-shell microcrystal with an epitaxial growth method. As for Eu^3+^ ions under 396 nm excitation, the energy is mainly absorbed and Eu^3+^ ions emit red luminescence. In response to 980 nm irradiation, Er^3+^ ions are excited and green emission are prominent. The photon luminescence emissions of the samples (β-NaYF_4_Yb/Er@β-NaYF_4_:Eu) in visible ranges are shown in Figure 5f. The red line and blue line are photoluminescence spectra of the microcrystals under excitation at 396 nm and 980 nm, respectively. The red line in Figure 5f contains three characteristic emission bands at 590 nm, 616 nm, and 690 nm. The emission wavelengths ascribe to the transition of D05→FJ7 (*J* = 1, 2, 4) of Eu^3+^. The green line shows the UC emission from Er^3+^/Yb^3+^. The obtained spectra in Figure 5 revealed that both processes don’t interact with each other in a single core-shell structure. The advantage of the samples would provide the possibilities for photovoltaic devices and anti-counterfeiting [45].

## 3. Materials and Methods 

### 3.1. Materials

All the chemicals are of analytical grade and used as received without further purification. Y(NO_3_)_3_·6H_2_O (99.99%), Lu(NO_3_)_3_·6H_2_O (99.99%), Yb(NO_3_)_3_·5H_2_O (99.99%), Er(NO_3_)_3_·6H_2_O (99.99%), Tm(NO_3_)_3_·6H_2_O (99.99%), Eu(NO_3_)_3_·6H_2_O (99.99%), NH_4_F, and NaF were supplied by HWRK Chemical Co. Ltd., Beijing, China. NaOH (>98%), ethylenediaminetetraacetic acid disodium salt (EDTA-2Na), oleic acid (OA), and ethanol were supplied by Aladdin Chemical Reagent Co. Ltd., Shanghai, China.

### 3.2. Preparation of β-NaYF_4_:Er/Yb Microrods

The β-NaYF_4_:Yb/Er microrods were synthesized by a hydrothermal reaction with oleic acid as a chelating agent. NaOH (0.75 g) was added into mixed solution containing 3.75 mL of deionized water (DI), 12.5 mL of ethanol and 12.5 mL of oleic acid. This solution was vigorously stirred, followed by the addition of an aqueous solution of NH_4_F (2 M; 2.5 mL) and 5 mL of rare-earth aqueous solution of Y(NO_3_)_3_ (Yb^3+^/Er^3+^:20/2 mol %, yellow color, Y; 0.2 M), (Yb^3+^/Er^3+^:5/0.05 mol %, green color, G; 0.2 M), (Yb^3+^/Er^3+^:80/2 mol %, red color, R; 0.2 M) to form a colloidal suspension. After 40-min of vigorous stirring, the suspension was transferred into a 50-mL Teflon-lined autoclave and heated to 220 °C for 12 h before cooling down to room temperature. The product was isolated by centrifugation and washed 3 times with DI and ethanol. The samples were obtained after drying at 70 °C for 12 h.

### 3.3. Preparation of Seeding Microrods

All the synthesized β-NaYF_4_:Yb/Er microrods could be used as seeds for epitaxial growth studies. Firstly, we dissolved β-NaYF_4_:Yb/Er microrods (0.03 mmol) in cyclohexane solution under ultrasonic treatment conditions. Then, the solution was centrifuged at 6500 rpm to precipitate the microrods. Secondly, the microrods were mixed with 4 mL of HCl solution (2 M) and 1 mL of ethanol [12]. Then, the mixture was centrifuged at 8000 rpm to remove surface capping ligands. Finally, the microrods were washed with ethanol for 2 times and then dissolved in 1 mL of DI as standby seeding.

### 3.4. Sequential Growth of Core-Shell Microrods

The core-shell structured β-NaYF_4_ microrods were synthesized by a hydrothermal reaction with EDTA-2Na as a chelating agent. Typically, 4.7 mL of the EDTA-2Na solution was mixed with the rare-earth aqueous solution Ln(NO_3_)_3_ (0.5, 0.8, 1.0, 1.5, 1.875 mL, 0.2 M; Ln = Y^3+^, Lu^3+^, Eu^3+^) under the condition of vigorous stirring and then NH_4_F (5 mL, 2 M), NaF (10 mL, 2 M), HCl (1.875 mL, 2 M) and HNO_3_ (1.875 mL, 15 wt %) were mixed to form the suspension. The resulting mixture was transferred into a 50-mL Teflon-lined autoclave, heated at 220 °C for 12 h and then cooled down to room temperature. The samples were respectively centrifuged and washed with DI and ethanol for 3 times.

### 3.5. Characterization

The structures of the samples were confirmed by power X-ray diffraction (XRD) in the 2*θ* ranging from 10 to 80 degrees by the D/max-2500 X-ray diffractometer (Riagaku Co. Ltd., Tokyo, Japan). The morphology of the products was observed under a scanning electron microscopy (SEM) (JSM-7500F, JEOL Ltd., Tokyo, Japan). The elemental mapping and line scanning were performed by EDS (JEOL Ltd., Tokyo, Japan), high-angle annular dark field imaging in the scanning transmission electron microscopy image (STEM). The UC emission spectra of the samples were recorded by a HORIBA Fluorolog-3 luminescence spectrometer (Horiba Jobin Yvon, Edison, NJ, USA) under a 980 nm laser with an optic fiber accessory. All the measurements were performed at room temperature.

## 4. Conclusions

We synthesized the uniform core-shell structured β-NaLnF_4_@β-NaLnF_4_ (Ln = Y, Lu, Yb) microcrystals via the epitaxial growth technique based on the hydrothermal reaction. The NaLnF_4_-based (Ln = Y, Yb, Lu, Gd) core-shell upconversion microcrystals provided a platform for the spatially confining optical process while possessing high luminescence efficiency. STEM and EDS analysis illustrated that the chemical composition distribution of the microcrystals was consistent with the designed element distribution, indicating the formation of core-shell structured microcrystals. The thickness of the shell can be controlled by adjusting the amount of shell precursor. It was found that the length of the β-NaYF_4_:Yb/Er@β-NaLuF_4_:Yb/Tm microcrystals increased from 1.87 μm to 2.70 μm and the diameter of the microcrystals increased from 0.3 μm to 0.42 μm after increasing the volume of the shell precursor solution from 0.5 mL to 1.875 mL (2 M). The thickness of the shell in our core-shell microcrystals has a great effect on the intensity of the shell dopant ions emission. But, the emission intensity of dopant ions in core (Er^3+^) does not change greatly with the shell thickness increasing. Furthermore, in the β-NaYF_4_:Yb/Er@β-NaLuF_4_:Yb/Tm system, the log—log plots of emission intensity versus excitation power density indicate that the plots of emissions peaks (475 nm, 542 nm and 647 nm) are nearly parallel, which means that it doesn’t exist complicated interaction among dopant ions. Meanwhile, the relative intensities of blue (475 nm), green (542 nm) and red (647 nm) emissions are nearly constant at different excitation power density, indicating that the emission color is independent on the power density. The core-shell microcrystals realized the color tuning and dual-mode emission at the single-particle level by incorporating a serious of lanthanide ions at specific concentrations into core and shell layer. The emission fluorescence would extend to almost the whole visible spectral region. The obtained core-shell microcrystals may be of great potential in anti-counterfeiting and photovoltaic applications.

## Supplementary Materials

The followings are available online at http://www.mdpi.com/2079-4991/7/12/448/s1, Figure S1: Scanning electron microscopy image of the as-prepared NaYF_4_:Yb/Er nanocrystals coating OA, Figure S2: Scanning electron microscopy image of NaYF_4_:Yb/Er seeding nanocrystals after removing surface capping ligands, Figure S3: Scanning electron microscopy image of NaYF_4_:Yb/Er seeding nanocrystals after coating shell, Figure S4: Investigation of core-shell structured NaYF_4_:Yb/Er (20/2 mol %)@NaYbF_4_ microcrystals, Figure S5: Investigation of core-shell structured NaYF_4_:Yb/Er (20/2 mol %, Y)@NaYF_4_:Yb/Er (80/2 mol %, R) microcrystals, Figure S6: Investigation of core-shell structured NaYF_4_:Yb/Er (20/2 mol %)@NaLuF_4_:Yb/Tm (20/0.2 mol %) microcrystals, Figure S7: Investigation of NaYF_4_:Yb/Er (20/2 mol %, Y)@NaLuF_4_:Yb/Tm (20/0.2 mol %) microcrystals growth versus the shell precursor content, Figure S8: Size distribution analysis of the NaYF_4_:Yb/Er (20/2 mol %, Y)@NaLuF_4_:Yb/Tm (20/0.2 mol %) microcrystals collected at various shell precursor contents, Figure S9: Investigation of core-shell structured NaYF_4_:Yb/Er (5/0.05 mol %)@NaYF_4_:Eu (10 mol %) microcrystals, Scheme SI: Schematic for core-shell structured β-NaLnF_4_ @β-NaLnF_4_ microcrystals.
