# Supplementary material for: Sequential Growth of Uniform β-NaYF4@β-NaLnF4 (Ln = Y, Lu, Yb) Microcrystals with Luminescent Properties of Multicolor Tuning and Dual-Mode Emission
Source: Nanomaterials (Basel). 2017 Dec 14;7(12):448. doi: 10.3390/nano7120448 (PMC5746938; doi:10.3390/nano7120448)
Supplement: Supplementary file 1 [file nanomaterials-07-00448-s001.pdf]

Sequential Growth of Uniform β-$NaYF_4$ @β-$NaLnF_4$ (Ln=Y, Lu, Yb) Microcrystals with Luminescent Properties of Multicolor Tuning and Dual-mode Upconversion (UC) /Downconversion (DC) Emission

Dandan Ju [1], Feng Song [1,2]*, Yingdong Han [1,2]*, Wenjing Cui [1,2], Aihua Zhou [1,2], Shujing Liu [3], Xueqin Wang [1,2], Ming Feng [1, 2] and Chengguo Ming [4]

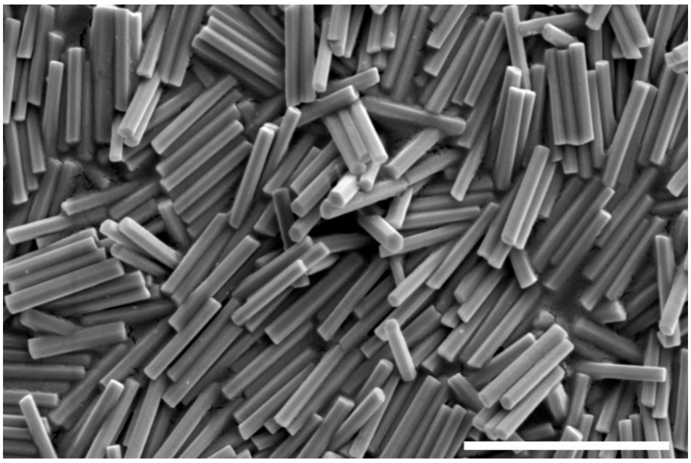

Figure S1. Scanning electron microscopy image of the as-prepared $NaYF_4$: Yb/ Er nanocrystals coating OA. Scale bar is 2 μm.

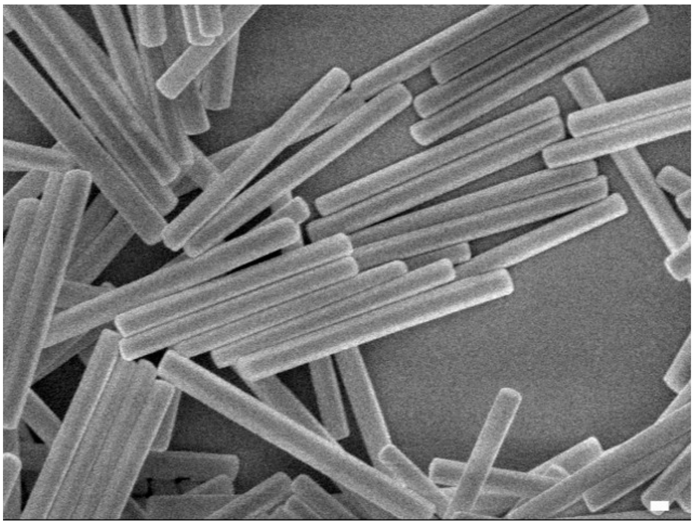

Figure S2. Scanning electron microscopy image of $NaYF_4$: Yb/Er seeding nanocrystals after removing surface capping ligands. Scale bar is 100 nm.

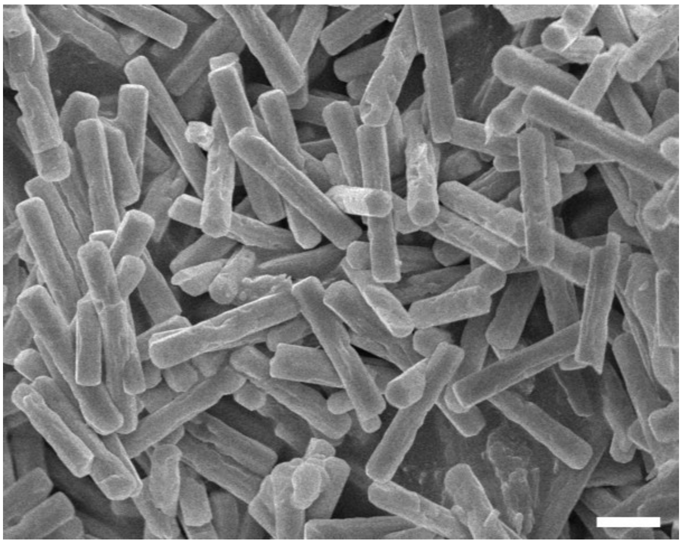

Figure S3. Scanning electron microscopy image of $NaYF_4$: Yb/Er seeding nanocrystals after coating shell. Scale bar is 1 μm.

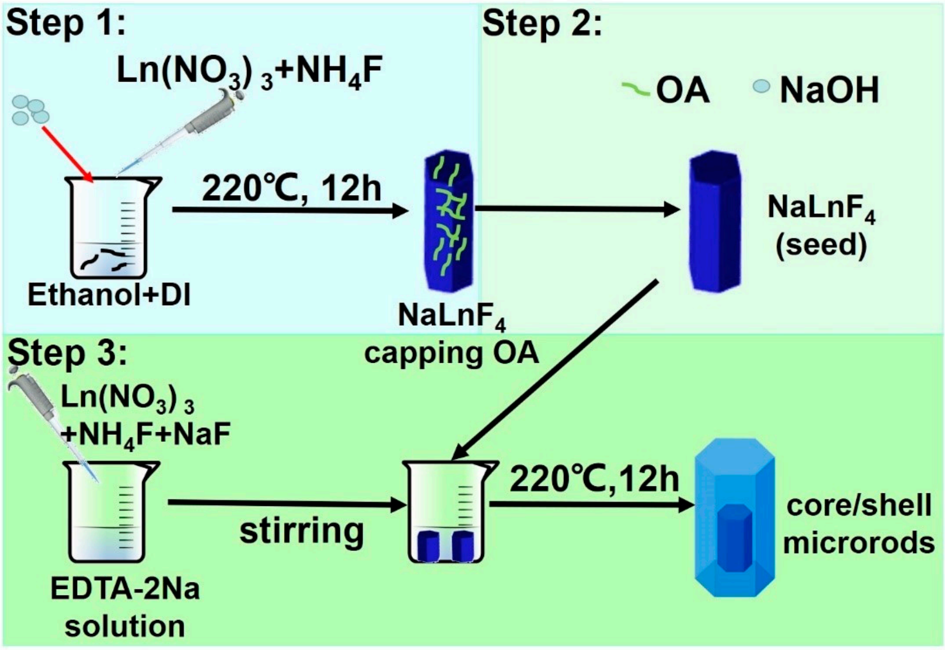

Scheme SI. Schematic for core-shell structured β-$NaLnF_4$@β-$NaLnF_4$ microcrystals.

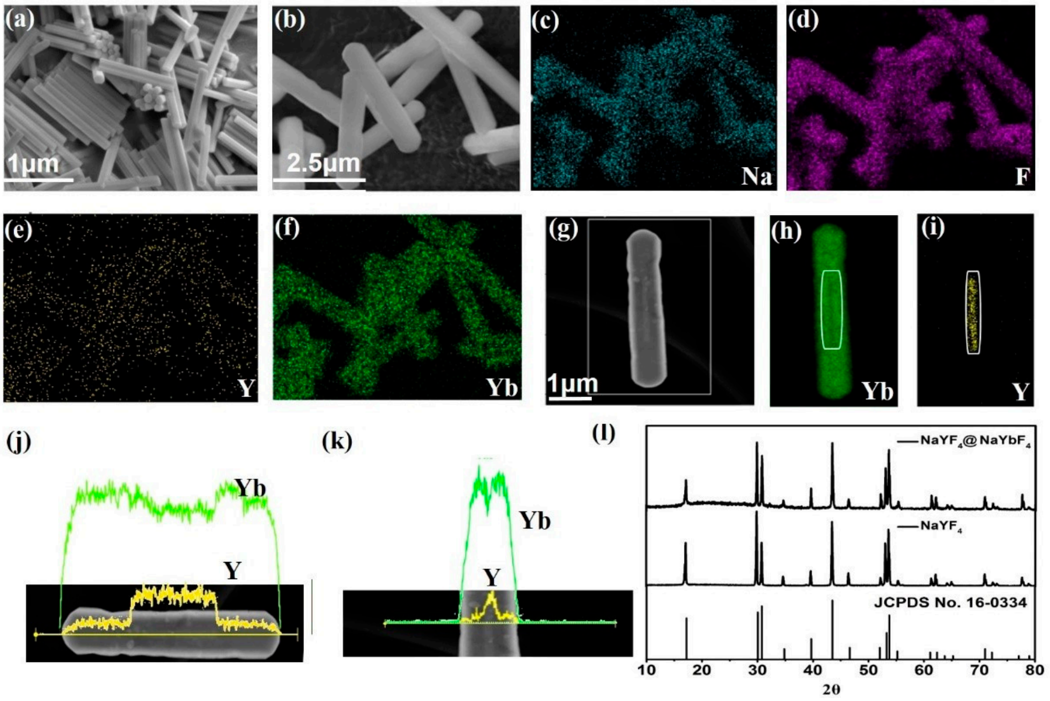

Figure S4. (**a**–**b**) SEM images of the microcrystals, $NaYF_4$: Yb/Er (20/2 mol%, Y) and $NaYF_4$: Yb/Er (20/2 mol%)@$NaYbF_4$. (**c**–**f**) Element mappings of Na, F, Y, and Yb in the microcrystals. (**g**) Scanning transmission electron microscopy image (STEM) of the $NaYF_4$:Yb/Er@$NaYbF_4$ microrods. (**h**–**i**) Element mappings of Yb and Y in a single core-shell microrod. The white boxes show the position of the core microrods. (**j**–**k**) Line scans of the elemental distribution in a heterogeneous single core-shell microrod. (**l**) XRD patterns of $NaYF_4$: Yb/Er microrods and $NaYF_4$:Yb/Er@$NaYbF_4$ microrods.

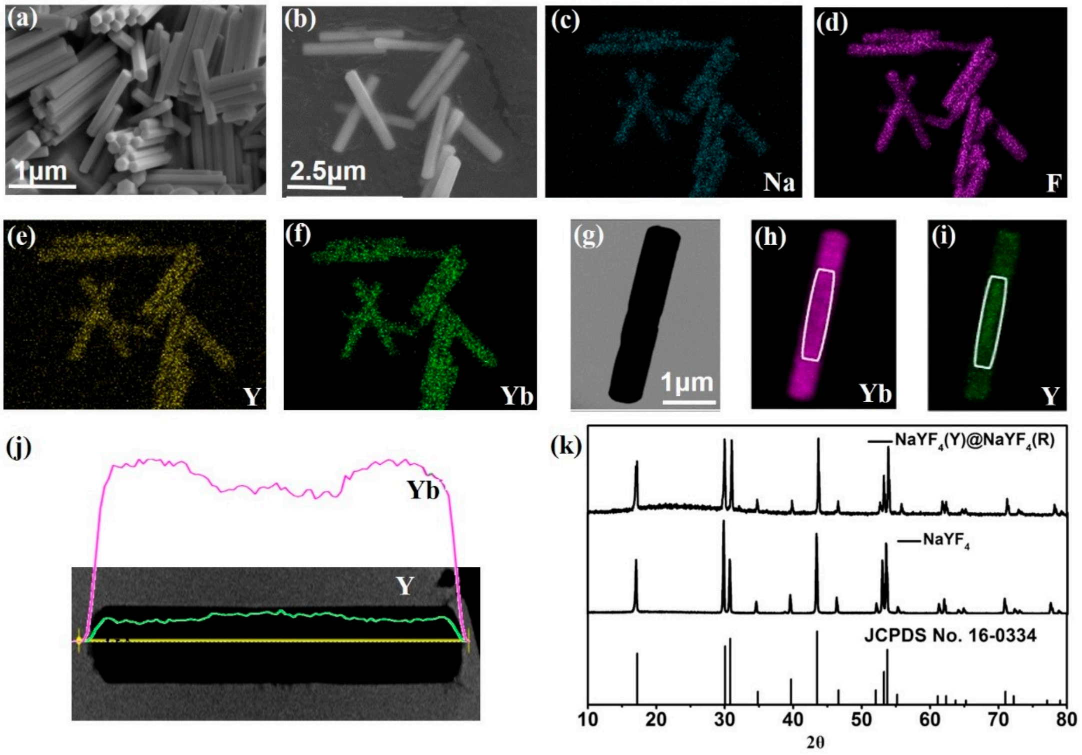

Figure S5. (**a**–**b**) SEM images of the microcrystals, $NaYF_4$: Yb/Er (20/2 mol%) and $NaYF_4$: Yb/Er (20/2 mol%, Y) @$NaYF_4$: Yb/Er (80/2 mol%, R). (**c**–**f**) Element mappings of Na, F, Y, and Yb in the microcrystals. (**g**) Scanning transmission electron microscopy image (STEM) of the $NaYF_4$:Yb/Er@

$NaYF_4$:Yb/Er microrods. (**h**–**i**) Element mappings of Yb and Y in a single core-shell microrod. The white boxes show the position of the core microrods. (**j**) Line scans of the elemental distribution in a single homogeneous core-shell microrod. (**k**) XRD patterns of $NaYF_4$: Yb/Er microrods and $NaYF_4$: Yb/Er (20/2 mol%, Y) @$NaYF_4$: Yb/Er (80/2 mol%, R) microrods.

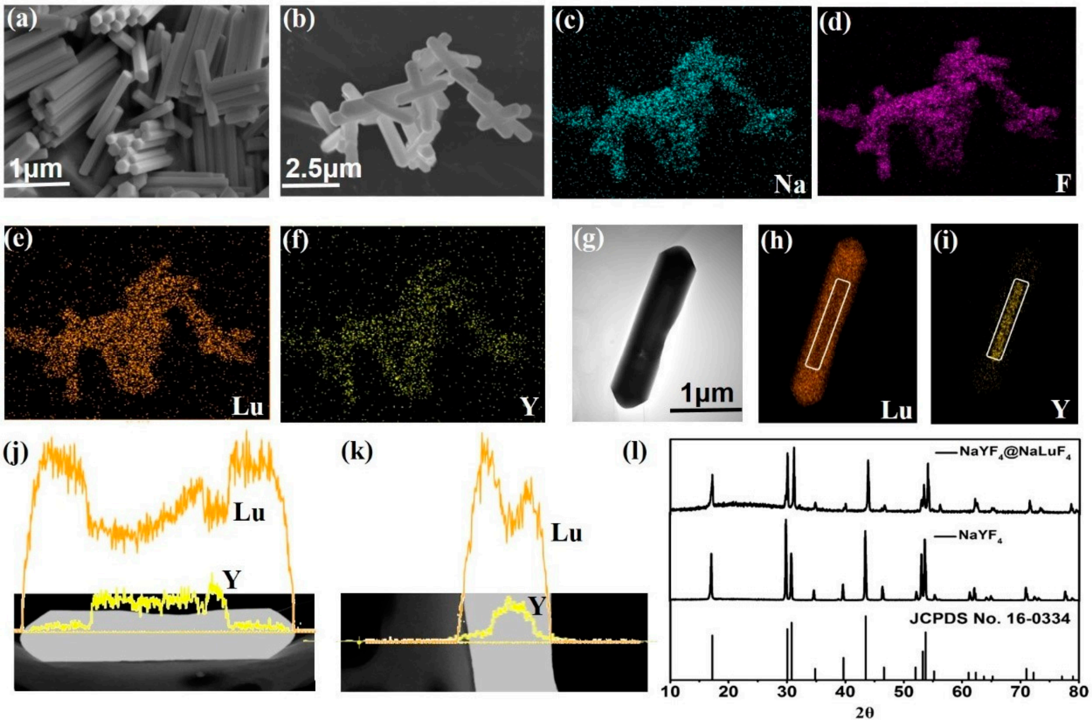

Figure S6. (**a**–**b**) SEM images of the microcrystals, $NaYF_4$: Yb/Er (20/2 mol%) and $NaYF_4$: Yb/Er (20/2 mol%) @ $NaLuF_4$: Yb/Tm (20/0.2 mol%). (**c**–**f**) Element mappings of Na, F, Lu, and Y in the microcrystals. (**g**) STEM of the core-shell microrods. (**h**–**i**) Element mappings of Lu and Y in a single core-shell microrod. The white boxes show the position of the seed. (**j**–**k**) Line scans of the elemental distribution in a single core-shell microrod. (**l**) XRD patterns of $NaYF_4$: Yb/Er microrods and $NaYF_4$:Yb/Er@$NaLuF_4$:Yb/Tm microrods.

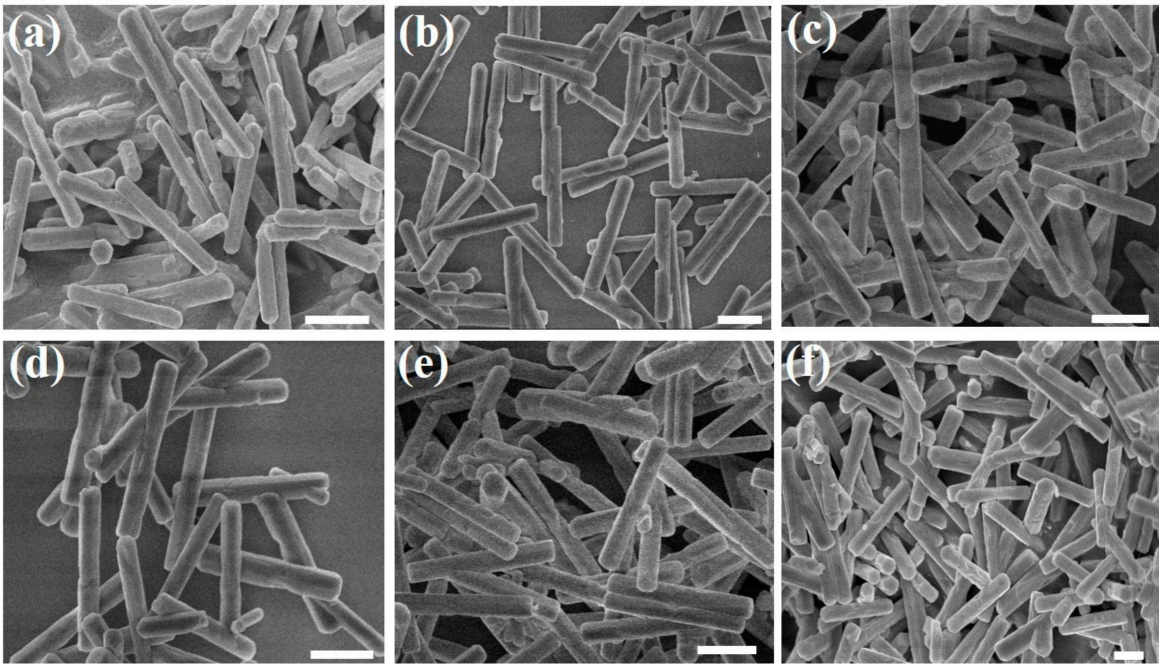

Figure S7. Investigation of $NaYF_4$: Yb/Er (20/2 mol%, Y) @$NaLuF_4$: Yb/Tm (20/0.2 mol%) microcrystals growth against the shell precursor content. (**a**–**f**) are the SEM images of the core-shell microcrystals with different shell thinkness, the shell precursor: (**a**) 0.5 mL, (**b**) 0.8mL, (**c**) 1.0 mL, (**d**) 1.2 mL, (**e**) 1.5 mL, (**f**) 1.875mL. (Scale bar: 1μm).

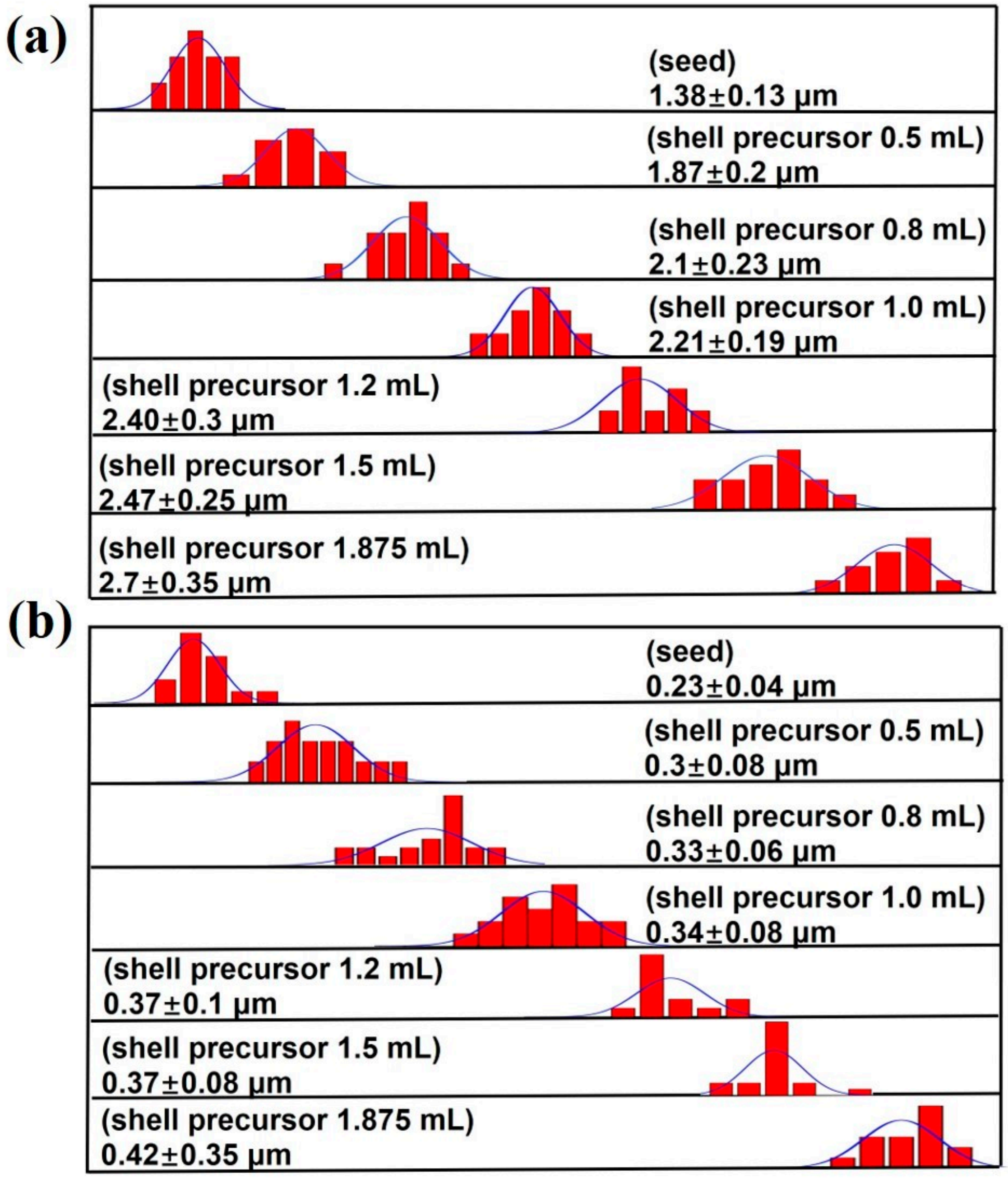

Figure S8. Size distribution analysis of the $NaYF_4$: Yb/Er (20/2 mol%, Y) @$NaLuF_4$: Yb/Tm (20/0.2 mol%) microcrystals collected at various shell precursor contents added.

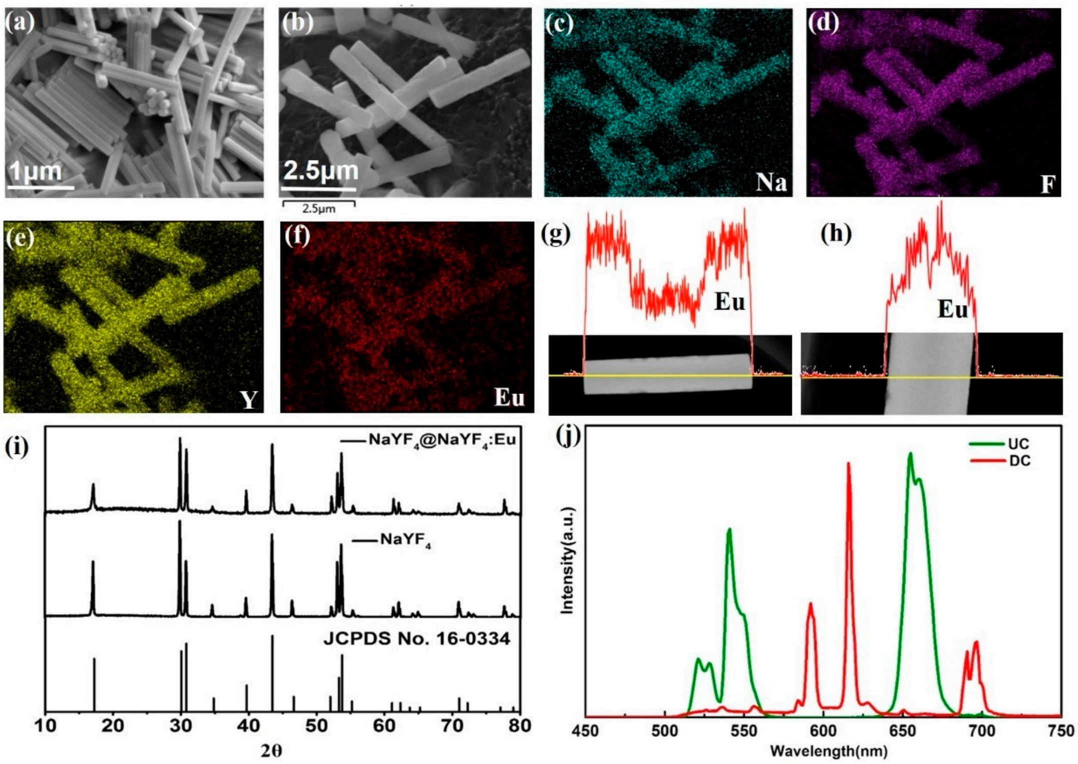

Figure S9. (**a**–**b**) SEM images of the microcrystals, $NaYF_4$: Yb/Er (5/0.05 mol%) and $NaYF_4$: Yb/Er (5/0.05 mol%)@ $NaYF_4$: Eu (10 mol%). (**c**–**f**) Element mappings of Na, F, Y, and Eu in the microcrystals. (**g**–**h**) Line scans of the elemental distribution in a single core-shell microrod. (**i**) XRD patterns of $NaYF_4$: Yb/Er microrods and $NaYF_4$:Yb/Er@$NaYF_4$:Eu microrods. (**j**) Emission spectra of the microcrystals under excitation at 396 nm. The green line and red line are emission spectra of $NaYF_4$:Yb/Er and $NaYF_4$: Yb/Er@$NaYF_4$:Eu, respectively.

1. Abel, K.A.; Boyer, J.C.; Andrei, C.M.; van Veggel, F. C. J. M. Analysis of the Shell Thickness Distribution on $NaYF_4$/$NaGdF_4$ Core/Shell Nanocrystals by EELS and EDS. *J. Phys. Chem. Lett.* 2011, **2**, 185-189. DOI: 10.1021/jz101593g.
2. Li, X.; Guo, Z.; Zhao, T.; Lu, Y.; Zhou, L.; Zhao, D.; Zhang, F.; Li, X. Filtration Shell Mediated Power Density Independent Orthogonal Excitations-Emissions Upconversion Luminescence. *Angew. Chem. Int. Ed. Engl.* 2016, **55**, 2464-2469. DOI: 10.1002/anie.201510609.

0.4, (**e**) 0.6, (**f**) 0.8, where $\varepsilon_0 = 8.85\times10^{-12}$, $\varepsilon_r = 10$, L = 160 nm, $V_{bi} + V_s = 0.3$ V.
